# Integrative Analysis of Liver Metabolomics and Transcriptomics Reveals Oxidative Stress in Piglets with Intrauterine Growth Restriction

**DOI:** 10.3390/biology11101430

**Published:** 2022-09-29

**Authors:** Hongmei Gao, Xiaoyou Chen, Junxing Zhao, Zhenhua Xue, Longchao Zhang, Fuping Zhao, Bingyuan Wang, Lixian Wang

**Affiliations:** 1 Institute of Animal Science, Chinese Academy of Agricultural Sciences, Beijing 100193, China; 2 College of Animal Sciences, Shanxi Agricultural University, Jinzhong 030801, China; 3 The Municipal Animal Husbandry General Station of Beijing, Beijing 100107, China

**Keywords:** intrauterine growth restriction (IUGR), piglet, liver, metabolomics, transcriptomics

## Abstract

**Simple Summary:**

Although the correlation between oxidative stress and liver metabolic dysfunction has been established in piglets with intrauterine growth restriction, detailed information concerning the molecular mechanisms that facilitate this relationship remains limited. In the present study, metabolomic and transcriptomic techniques were used to investigate differences in gene expression and metabolites in the liver of piglets in intrauterine growth restriction and normal birth weight groups to systematically analyze the potential mechanism of metabolic characteristics induced by oxidative stress in the liver of intrauterine growth restriction piglets. Our results revealed that the intrauterine growth restriction piglets were involved in a variety of metabolic abnormalities, including mitochondrial dysfunction, imbalance of fatty acid composition, disruption to sources of one-carbon unit supply, and abnormal galactose conversion, which may be responsible for oxidative stress in the liver.

**Abstract:**

The correlation between oxidative stress and liver metabolic dysfunction in piglets with intrauterine growth restriction (IUGR) remains limited. Therefore, the objective of the present study was to investigate potential mechanisms of metabolic characteristics induced by oxidative stress in the livers of IUGR piglets using metabolomic and transcriptomic analysis. Analysis of the phenotypic characteristics showed that the liver weight of the intrauterine growth restriction piglets was significantly lower than that of normal birth weight piglets. Intrauterine growth restriction piglets exhibited disordered hepatic cord arrangement and vacuolization as well as excessive lipid accumulation in hepatocytes. In addition, the activities of antioxidant enzymes were significantly decreased in the liver of the intrauterine growth restriction piglets, whereas the level of the lipid peroxidation marker MDA was significantly increased. Finally, our findings revealed that intrauterine growth restriction piglets were involved in a variety of metabolic abnormalities, including mitochondrial dysfunction, imbalance of fatty acid composition, disruption to sources of one-carbon unit supply, and abnormal galactose conversion, which may be responsible for oxidative stress in the liver. In summary, these data provided a detailed theoretical reference for revealing the hepatic metabolic characteristics of intrauterine growth restriction piglets.

## 1. Introduction

Piglet mortality is an ongoing concern in global pork production. The first week after birth is the most critical for piglet survival due to the higher risk of death during this period [1]. Low birth weight is likely to be an important determinant of the high mortality rate of neonatal piglets as low birth weight piglets usually die from crushing, weakness, starvation, and other congenital defects within the first few days of life [2]. Most low birth weight piglets are recognized as intrauterine growth restriction (IUGR) piglets. They usually suffer from intrauterine crowding and placental inefficiency, which stunts fetal growth potential [3]. IUGR in piglets not only shows higher mortality and morbidity in the short term but is also related to disturbances in metabolism, stunted growth, and poor carcass quality [4]. Considerable evidence suggests that oxidative stress is a major contributor to metabolic disorders in various IUGR models. Some studies have shown that a suboptimal prenatal environment can trigger fetal oxidative stress, which may be the main cause of metabolic organ dysfunction, resulting in inflammation, apoptosis, and autophagy, ultimately impairing fetal growth [5]. However, detailed information regarding oxidative stress leading to metabolic abnormalities in individuals with IUGR remains limited.

The liver plays a central role in the management of systemic homeostasis by regulating energy metabolism; therefore, liver dysfunction may cause widespread metabolic disorders. Previous studies have shown that individuals with IUGR exhibit hepatic dysfunction [6,7]. In piglets, IUGR alters the expression of genes associated with hepatic energy production and mitochondrial biogenesis, which impairs hepatic mitochondrial oxidative phosphorylation and reduces hepatic mitochondrial DNA content [8,9,10]. In addition, compromised utilization of circulating triglycerides and less optimized lipid metabolism have been reported in IUGR piglets [11]. Our previous study revealed dysregulation of glucocorticoids in the liver of IUGR piglets [12]. We hypothesized that the liver metabolism of IUGR piglets would also be abnormal. It is well known that glucocorticoid secretion occurs mainly in response to cellular stress activities through the hypothalamic–pituitary–adrenal (HPA) axis [13]. Although the above reports have directly or indirectly established a negative correlation between oxidative stress and liver metabolic dysfunction in IUGR piglets, the molecular mechanisms that facilitate this relationship require further investigation.

Omics technology has accelerated the development of quantitative and high-throughput. Though integrating multiomics analysis, key molecular functions and metabolic pathways involved in complex biological processes can be explored in-depth [14]. In our previous study, we successfully conducted transcriptome sequencing of the livers of IUGR piglets [12]. Hence, in this study, we first applied metabolome to assess liver metabolic changes between IUGR and normal birth weight (NBW) groups. Second, we conducted an integrative analysis of metabolomics and transcriptomics to explore the potential mechanism of metabolic characteristics induced by oxidative stress in the liver of IUGR piglets.

## 2. Materials and Methods

### 2.1. Sample Collection

All animals were treated humanely according to the criteria outlined in the “Guide for the Care and Use of Laboratory Animals” published by the Institute of Animal Sciences, Chinese Academy of Agricultural Sciences (Beijing, China). The experimental protocols used in this study were approved by the Animal Care and Use Committee (IAS2021-65).

In total, 17 full-term neonatal piglets (Landrace sires × Large White dams) were recruited, including 9 IUGR piglets and 8 NBW controls. According to the criteria [15]. The body weight of IUGR piglets should be chosen less than two standard deviations within the average weight in the litter, whereas the body weight of NBW piglets should be within one standard deviation of the average weight. In addition, IUGR piglets were categorized based on their head morphological characteristics using a previously described method [16]: (1) steep-dolphin-like forehead, (2) bulging eyes, and (3) wrinkles perpendicular to the mouth. Piglets with at least one of the three characteristics were defined as IUGR.

Both the body and liver weights of the piglets were measured after sacrifice. Liver samples were collected, washed, and frozen in liquid nitrogen for further use.

### 2.2. Widely Targeted Metabolomics and Analysis

Metabolomics analyses were performed at MetWare Biotechnology Co., Ltd. (Wuhan, China) as previously described [17], including liver tissue extraction, metabolite identification, and quantification.

For each metabolite, Student’s *t*-test was used to assess the statistical significance of *p* values, and OPS-DA was used to determine the variable importance of projection (VIP) scores. Significantly differential metabolites between the groups were determined under the criteria: VIP ≥ 1, *p* < 0.05, and absolute Log_2_FC (fold change) ≥ 1. All analyses were performed using R statistical software V.3.6.1 (Vienna, Austria).

### 2.3. Bioinformatic Analysis of Transcriptome

RNA sequencing sources were collected from the Sequence Read Archive (Bio-Project: PRJNA597972; https://www.ncbi.nlm.nih.gov/bioproject/PRJNA597972 (accessed on 1 May 2022). Due to the update of the pig reference genome, the filtered sequence reads were realigned to the Sscrofa11.4 reference genome from the Ensembl annotation system. Bioinformatics analysis was performed as previously described [12] and integrative analysis was performed by MetaboAnalyst V.5.0 tools (McGill University, Quebec, QC, Canada; https://www.metaboanalyst.ca/ (accessed on 15 July 2022).

### 2.4. Detection of Oxidative Stress Biomarkers in Liver

Commercial kits for detecting the total antioxidant capacity (T-AOC), superoxide dismutase (SOD), glutathione peroxidase (GSH-Px), catalase (CAT), glutathione (GSH), and malondialdehyde (MDA) were purchased from the Nanjing Jiancheng Institute of Biotechnology (Nanjing, China), and liver tissue was processed and biochemically analyzed following to the manufacturer’s instructions.

### 2.5. Transmission Electron Microscopy Analysis

The procedure for liver hematoxylin eosin (H&E) and Oil Red O (ORO) staining was performed according to previous reports [18]. Liver tissue specimens were dehydrated, fixed, and stained with H&E or ORO dye. The histopathology of liver tissues was evaluated through a light microscope (Nikon ECLIPSE 80i; Nikon Corporation, Tokyo, Japan). Two independent observers performed a double-blind assessment and each was observed in at least three different viewpoints.

### 2.6. Liver Morphological Evaluation

Liver samples for transmission electron microscopy (TEM) were prepared according to previously described [19]. Briefly, the liver tissue was sequentially fixed in 2.5% glutaraldehyde and 1% osmium tetroxide, dehydrated, and finally embedded. Then the tissue was sliced into ultrathin sections (60–80 nm) and stained with 2% uranyl acetate and lead citrate, observed under TEM (Hitachi HT-7700, Tokyo, Japan).

### 2.7. RNA Isolation and Real-Time qPCR

Total RNA of liver was extracted using Invitrogen^TM^ TRIzol (Thermo Fisher Scientific, Carlsbad, CA, USA). cDNA was synthesized using PrimeScript™ RT reagent Kit (Takara, kusatsu, shiga, Japan). Real-time qPCR was performed in Bio-Rad fluorescence detection system (CFX96 RealTime PCR, Bio-Rad Laboratories, Hercules, CA, USA) as previously described [20]. Real-time qPCR was repeated three times independently and the 2^−ΔΔCt^ method was used to measure the relative mRNA expression of target genes [21]. GAPDH was a reference gene and the primers information are listed in Supplementary file: Table S1.

### 2.8. Statistical Analysis

Statistical analysis was performed using SPSS v.21.0 (IBM Headquarters, Chicago, IL, USA). The Student’s *t-*test was used to analyze the differences between the two groups. The data are presented as means ± standard deviation (SD), and *p* values are indicated by asterisks to indicate statistically significant differences (* *p* < 0.05; ** *p* < 0.01).

## 3. Results

### 3.1. Phenotypic Traits of Piglets in IUGR and NBW Groups

**Figure 1:**
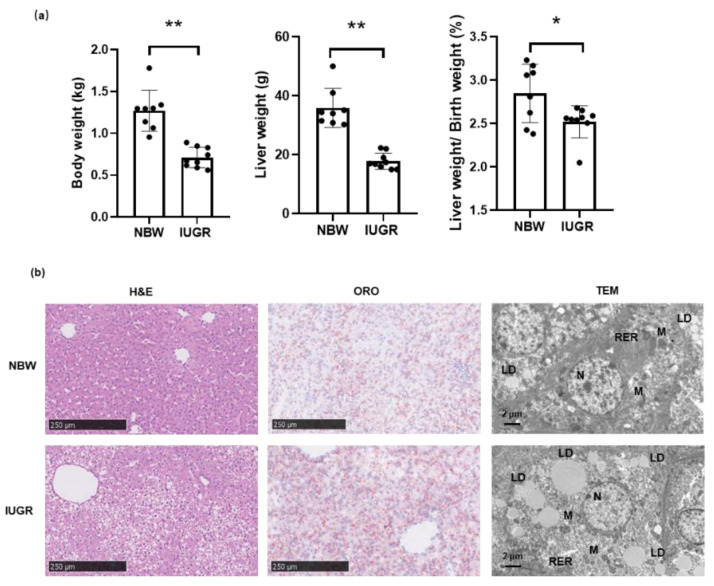
Phenotypic traits of piglets between IUGR and NBW group. (**a**) Body weight, liver weight, as well as the relative liver weight of the piglets. The data are presented as the means ± SD, and *p* values are indicated by asterisks to indicate statistically significant differences (* *p* < 0.05; ** *p* < 0.01). (**b**) Histopathological characteristics of liver in IUGR and NBW piglets, light microscopy of hematoxylin eosin (H&E) and Oil Red O (ORO) staining, as well as transmission electron micrographs (TEM). Abbreviations: N, nucleus; M: mitochondria; RER: rough endoplasmic reticulum; LD: lipid droplets.

The body weight of all piglets in this study are shown in Figure 1a. As expected, the initial body weight of newborn IUGR piglets was approximately 44% lower than that of NBW piglets. At the same time, both the absolute and relative weights of the liver were significantly lower in IUGR piglets than in NBW piglets.

Histopathological examinations of hepatic sections from IUGR and NBW piglets are presented in Figure 1b. The NBW group exhibited normal histomorphological structures. Conversely, apparent vacuolization and disordered arrangement of the hepatic cords were observed in the hepatocytes of IUGR piglets. Notably, ORO staining revealed increased lipid accumulation in the IUGR group. Furthermore, TEM showed significant pathological changes in the hepatocyte ultrastructure of IUGR piglets, consistent with our previous results that IUGR piglets have shorter, globular, and smaller mitochondria compared to the NBW group; in addition, a greater volume and number of lipid drops were observed in the cytoplasm.

### 3.2. Liver Cellular Stress in the Liver of Piglets

**Figure 2:**
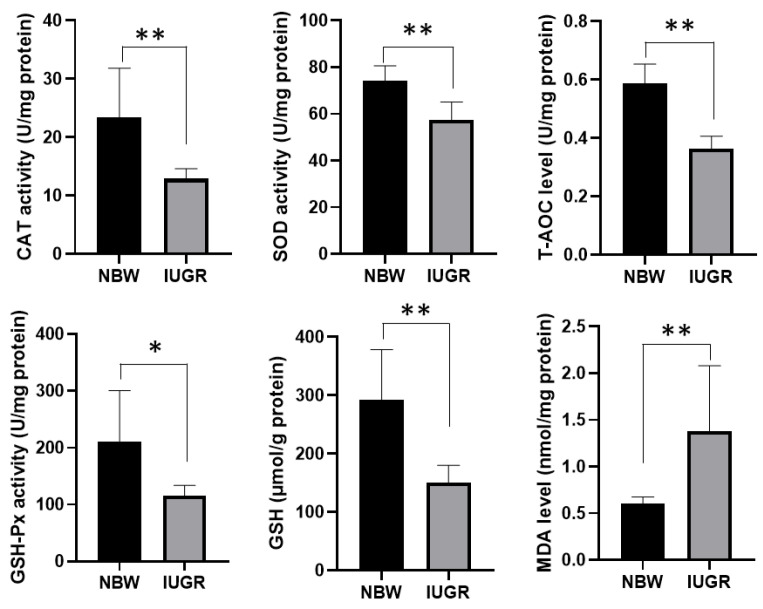
The activity of SOD, CAT, GSH-Px, T-AOC, MDA, and the concentration of GSH in liver between IUGR and NBW piglets. Differences were assessed by *t*-test (*n* = 8) and asterisks represent the *p* values (* *p* < 0.05; ** *p* < 0.01).

To explore hepatic oxidative stress in piglets, we examined the key antioxidant activities, including SOD, CAT, GSH-Px, T-AOC, as well as the level of GSH in liver tissues from IUGR and NBW groups. The antioxidative enzymes activities of SOD, CAT, T-AOC, GSH-Px, and the level of GSH in the liver of IUGR piglets were noticeably decreased compared to those in the NBW group. Meanwhile, the level of the lipid peroxidation marker MDA was significantly increased in the IUGR group compared with that in NBW group (Figure 2).

### 3.3. Liver Metabolic Profiles in IUGR and NBW Piglets

**Figure 3:**
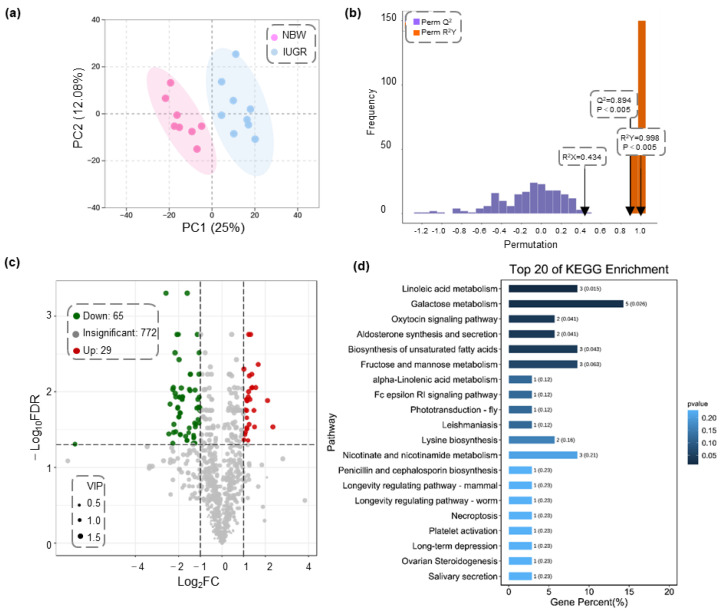
Metabolomics analysis of liver in the IUGR and NBW piglets. (**a**) Principal component analysis (PCA) scores plot derived from liver metabolomics between the NBW and IUGR group. (**b**) The permutation tests between NBW and IUGR group. (**c**) Volcano plot of DMs between NBW and IUGR piglets. Red, green, and gray dots indicate significantly upregulated, significantly downregulated, and non-significant metabolites, respectively. (**d**) The top 20 enriched KEGG pathways in the IUGR piglets.

To better explore the underlying mechanisms of liver metabolism in IUGR piglets, we employed a targeted UPLC−MS/MS approach for comprehensive metabolic profiling of the livers from the IUGR and NBW piglets. Based on metabolomics and multivariate statistical analyses, 866 known metabolites were detected in the IUGR and NBW groups. PCA score plots showed a clear separation trend (Figure 3a). Subsequently, an OPLS-DA model was constructed with one predictive and two orthogonal components (R^2^X = 0.434, R^2^Y = 0.998, Q^2^ = 0.894), based on the detected metabolites (Figure 3b). These results indicated that the liver metabolic profile of IUGR piglets is distinct from that of NBW controls, demonstrating satisfactory modeling and predictive abilities.

Significantly different liver metabolites (DMs) between IUGR and NBW piglets were selected under the criteria: VIP ≥ 1, *p* < 0.05, and absolute Log_2_FC ≥ 1. Ultimately, 94 DMs, including 65 downregulated and 29 upregulated DMs, were identified (Figure 3c; Supplementary file: Table S2). Among them, urobilin showed the highest decrease among the identified metabolites in IUGR piglets with Log_2_FC = −6.79, and L-theanine was the most increased metabolite with Log_2_FC = 2.35. KEGG analysis revealed that the DMs were significantly enriched in linoleic acid metabolism, galactose metabolism, oxytocin signaling pathway, aldosterone synthesis and secretion, and biosynthesis of unsaturated fatty acids (*p* ˂ 0.05), as shown in Figure 3d. Among the DMs involved in the above pathways, the levels of gamma-linolenic acid (GLA), arachidonic acid (AA, 20:4n-6), hydroxy octadecaenoic acids (HODEs; 9-HODE and 13-HODEs), eicosapentaenoic acid (EPA, 20:5n-3), and N-acetyl-D-galactosamine were significantly lower in the IUGR group than those in the NBW group. Conversely, the relative abundance of galactitol, inositol, sorbitol, cyclic ADP ribose (cADPR), and nicotinic acid adenine dinucleotide (NAD+) was higher in the IUGR group than in the NBW piglets.

### 3.4. Liver Transcriptomic Analysis of IUGR and NBW Piglets

**Figure 4:**
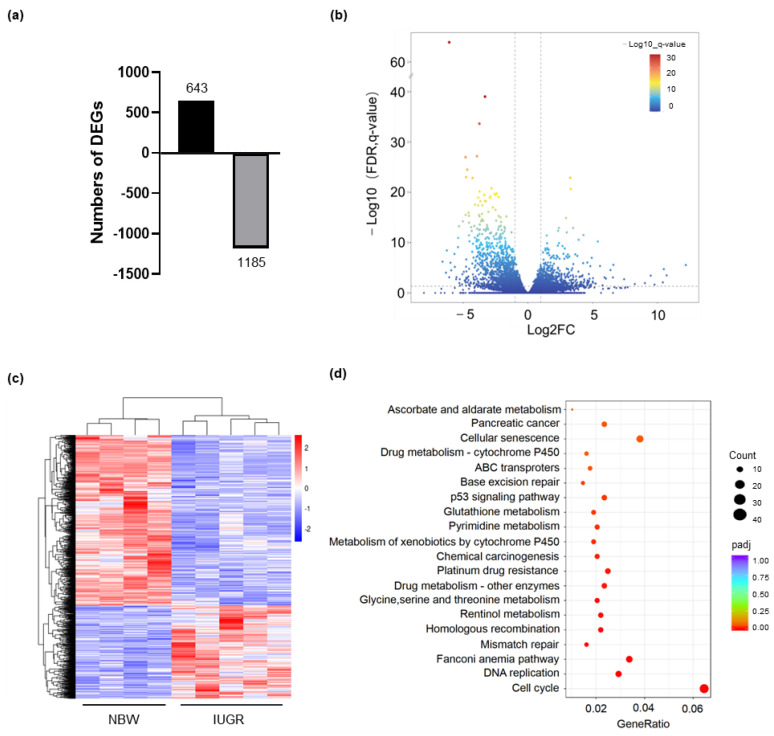
Bioinformatics analysis of liver transcriptome between the IUGR and NBW piglets. (**a**) The number of DEGs between the IUGR and NBW piglets. (**b**) Volcano plot of DEGs between the IUGR and NBW piglets. (**c**) The cluster heatmap between the IUGR and NBW piglets. (**d**) The top 20 enriched KEGG pathway terms were significantly altered in the IUGR piglets.

We selected five IUGR and four NBW piglet samples from the previously published RNA sequencing data of neonatal piglets for transcriptomic analysis, and screened out 1828 differentially expressed genes (DEGs) (*padj* ≤ 0.05; Fold Change ≥ 2 or ≤ 0.5), of which 643 were upregulated and 1185 were downregulated in the IUGR group (Figure 4a–c, Supplementary file: Table S3). Subsequently, we performed KEGG pathway enrichment analysis to explore the potential functions of these DEGs and displayed the top 20 enriched pathways in the IUGR group compared to the NBW group (Figure 4d). The top enriched pathways, such as glycine, serine, and threonine metabolism, glutathione metabolism, p53 signaling pathway, and cellular senescence, were all closely related to oxidative stress.

### 3.5. Integrative Analysis of Differential Transcriptomic and Metabolomic Profiles between IUGR and NBW Piglets

**Figure 5:**
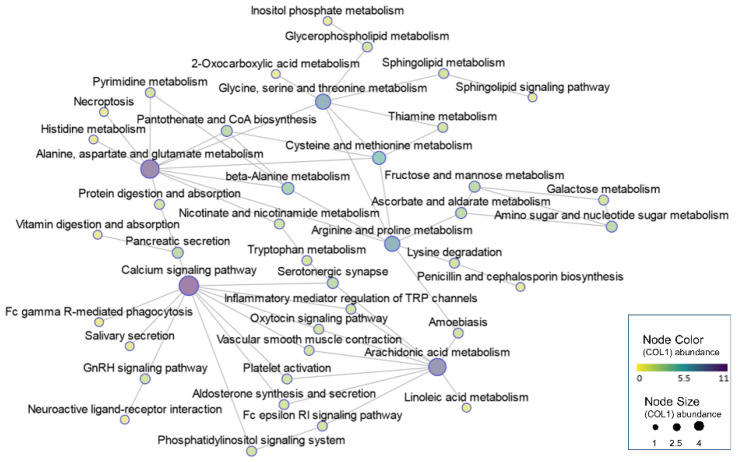
A network of interrelationships that integrate pathways in metabolomics and transcriptomics.

Given that the molecular mechanisms and metabolic pathways in the livers of IUGR piglets have not been fully elucidated, we conducted a comprehensive metabolome and transcriptome analysis to understand the regulatory network of hepatic metabolism in IUGR piglets. By mapping all DEGs and DMs to the KEGG pathway database, we obtained their common pathway in which both the DMs and DEGs were involved by joint pathway analysis. The interrelations of the integrated pathways were further obtained globally diagram using visual network analysis (Figure 5, Supplementary file: Table S4). Based on the integrated analysis, we further focused on the DEGs and DMs involved in these pathways, which were closely related to oxidative stress. These pathways were classified into four categories based on physiological processes, including mitochondrial dysfunction (nicotinate and nicotinamide metabolism, oxytocin signaling pathway, aldosterone synthesis and secretion, and calcium signaling pathways), imbalance of fatty acid composition (biosynthesis of unsaturated fatty acids, linoleic acid metabolism, alpha-linolenic acid metabolism, and arachidonic acid metabolism), disruption to sources of one-carbon unit supply (glycine, serine and threonine metabolism, and cysteine and methionine metabolism), as well as abnormal galactose conversion (galactose metabolism, fructose and mannose metabolism, and inositol phosphate metabolism) (Table 1).

### 3.6. The Validation of DEGs Expression Involved in the Integrated Pathways between IUGR and NBW Piglets

**Figure 6:**
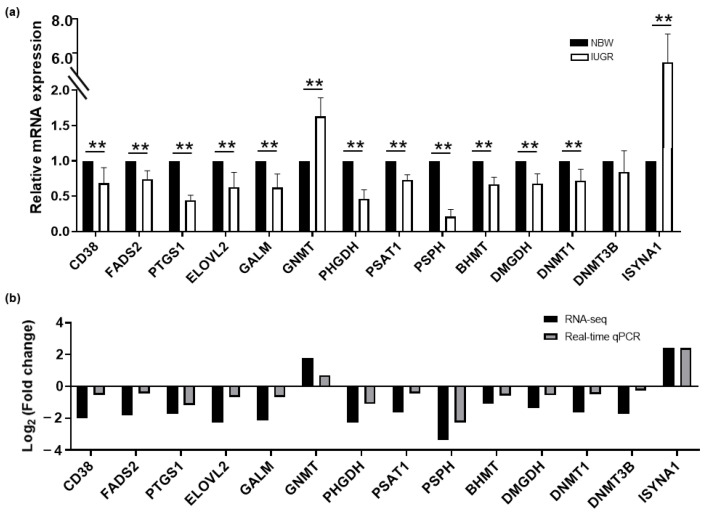
Verification of DEGs expression involved in the integrated pathways between IUGR and NBW piglets. (**a**) The relative expression of mRNA was determined by real-time qPCR. Results were presented as means ± SD. Differences were assessed by *t*-test (*n* = 8) and asterisks represent the *p* values (** *p* < 0.01). (**b**) log_2_Foldchange of selected genes between real-time qPCR and RNA-seq analysis.

We selected several key DEGs involved in the integrated pathways that may directly or indirectly regulate oxidative stress in the liver of IUGR piglets and to validate their expression patterns. The results showed that the mRNA expressions of *NNMT*, *CAMK1D*, *CACNB1*, *GNMT,* and *ISYNA1* were significantly increased in IUGR piglets, whereas the mRNA expressions of *CD38*, *BST1*, *CYP2C*, *FADS2*, *PTGS1*, *ELOVL2*, *ACOT7*, *GALM*, *PHGDH*, *PSAT1*, *PSPH*, *BHMT*, *DMGDH*, and *DNMT1* were significantly increased in the NBW group (Figure 6a). Moreover, the similar pattern for the expression of all genes was found between the real-time qPCR and our previous RNA-seq data (Figure 6b).

## 4. Discussion

According to Barker’s ‘thrifty phenotype’ hypothesis, liver growth is impaired in IUGR individuals, as indicated by a decrease in abdominal circumference at birth [22]. Consistent with this, we observed similar results in our previous and current study [12,23], as both the absolute and relative weight of the liver were significantly lower in IUGR piglets. Combined with our previous report, IUGR piglets undergo rapid catch-up growth in the early postnatal period; this may be due to the malnourished liver adapting to the abundant postnatal environment, and gaining excessive growth relative to other organs, which may in turn be detrimental to hepatic function, further exacerbating risk of metabolic syndrome [24].

Previous studies have revealed that mitochondrial dysfunction in the liver often causes increased levels of superoxide radical metabolites, which may lead to oxidative damage. Mitochondria are abundant in the liver, and when mitochondria are damaged or dysfunctional, the liver is bound to suffer from oxidative stress [25]. Similarly, many studies have revealed mitochondrial stress in the liver of IUGR individuals [8,9,10,26]. In aerobic respiration, mitochondria use oxygen as the terminal electron acceptor; however, this process produces damaging reactive oxygen species (ROS). Oxidative stress occurs when an imbalance appears between ROS and antioxidant enzymes, which leads to the transformation of ROS into less harmful molecules [27]. NAD^+^ is an enzyme cofactor, and its metabolites are key regulators of mitochondrial oxidative metabolism and stress resistance [28]. Recent studies have shown that cADPR, an important metabolite of NAD^+^, is decomposed by the NAD^+^-dependent enzyme *CD38*, which has been proposed as a second messenger that regulates intracellular calcium levels in various cellular processes [29]. In this study, compared with the NBW group, abnormal expression of NAD^ +^-consuming enzymes (*CD38*, *SIRT4*, *BST1*, and *NNMT*) and calcium signaling-related genes (*CAMK1D*, *CAMK1*, *CALML4*, *CACNB1*, and *CACNB3*), as well as altered concentrations of NAD+, N-methyl-2-pyridone-5-carboxamide, and cADPR were observed in the IUGR group. Meanwhile, the above DEGs and DMs were involved in nicotinate and nicotinamide metabolism, aldosterone synthesis and secretion, as well as the oxytocin signaling pathway, which were significantly enriched in the IUGR group. Recent evidence suggests that oxytocin signaling and the renin–angiotensin–aldosterone system (RAAS) are closely related to glucose uptake and lipid utilization, whereas dysfunction of oxytocin and the RAAS may lead to ROS imbalance and contribute to the pathogenesis of insulin resistance and dyslipidemia [30,31]. Therefore, the molecular mechanism of the dysregulated intracellular NAD^+^ levels observed in the livers of IUGR piglets may involve the disruption of calcium homeostasis, leading to the excess production of ROS.

Furthermore, in this study, abnormal regulation of fatty acids was observed in IUGR piglets. Linoleic acid (LA) metabolism and unsaturated fatty acid biosynthesis pathways were significantly enriched in the IUGR group, coupled with a decreased concentration of long-chain (≥C20) polyunsaturated fatty acids (LC-PUFA), such as EPA and AA. Long-chain PUFA is an important component of all cell membranes, especially AA and EPA, which are important precursors for the synthesis of first messengers and eicosapanoic acid compounds, and an imbalance in the n-3/n-6 ratio in LC-PUFAs is associated with metabolic syndrome [32]. Moreover, AA and its primary metabolites play an important role in the pro-inflammatory process and increase the ROS generation [33]. EPA is involved in reducing lipid accumulation and alleviating pathology in the liver [34]. In addition, LA and α-linolenic acid (ALA) are precursors for the synthesis of LC-PUFA. The synthesis process from PUFA to LC-PUFA needs the synergistic activities of fatty acid desaturase (FADS) and elongase (ELOVL) [35,36]. Moreover, alterations involving the LC-PUFA synthesis pathway causes an imbalance in the n-3/n-6 ratio and lipid peroxidation, which also leads to the production of ROS, subsequently damaging cellular functions [37]. Previous studies have found that abnormal expression of *ELOVL2* and *FADS2* in liver may lead to membrane fatty acid remodeling and impaired membrane lipid homeostasis [38]. In this result, the mRNA expression levels of *ELOVL2*, *FADS2*, *ACOT7*, *CYP2C*, *CYP4A*, *PTGS1*, *ALOX5*, and *GGT5*, which are involved in EPA and AA metabolism, were also altered in the livers of IUGR piglets. In addition, we also found increased hepatocyte lipid droplets in IUGR piglets, combined with av increased triglyceride content as reported in previous studies [12], which may be due to the imbalance of the n-3/n-6 ratio. Moreover, as the final products of lipid peroxidation, MDA is the main marker of superoxide free radicals [39]. High concentration of MDA was observed in the livers of IUGR piglets, suggesting that fatty acid metabolism disorder contributes to excessive oxidized stress in the liver of IUGR piglets.

Compared with those in the NBW group, the levels of SOD, CAT, GSH-Px, T-AOC, and GSH, all of which are antioxidant enzymes that eliminate ROS, were significantly decreased in the IUGR group, suggesting that the antioxidative abilities of the liver in IUGR piglets were impaired. In particular, GSH is an important scavenging molecule synthesized from glutamate, glycine, and cysteine, which protects cells from oxidation and maintains redox homeostasis [40]. From our results, the DEGs and DMs involved in glycine, serine, and threonine metabolism, as well as cysteine and methionine metabolism, were significantly enriched in the IUGR group. These metabolic pathways are involved in the supply of carbon sources in one-carbon metabolism and GSH production. In these pathways, the gene expression levels involved in de novo serine synthesis (*PHGDH*, *PSAT1*, and *PSPH*), and the glycine cleavage system (*GNMT*, *ALAS2*, *DMGDH*, *BHMT*, *GATM*, *GCAT*, and *AGXT2*), as well as dimethylglycine (DMG), were dysregulated in the livers of IUGR piglets. In addition, serine metabolism fuels the methionine salvage pathway, which is the main mechanism of synthesizing of the methyl donor S-adenosyl methionine (SAM). Subsequently, SAM provides methyl groups for epigenetic methylation and is metabolized to homocysteine (Hcy), which can enter the trans-sulfuration pathway to produce cysteine and finally GSH [41]. According to our results, the DNA methyltransferases *DNMT1* and *DNMT3b* were also downregulated in the livers of IUGR piglets. Therefore, all the dysregulation of DGEs and DMs involved in the one-carbon cycle in IUGR piglets may be one mechanism by which IUGR influences hepatic GSH production.

**Figure 7:**
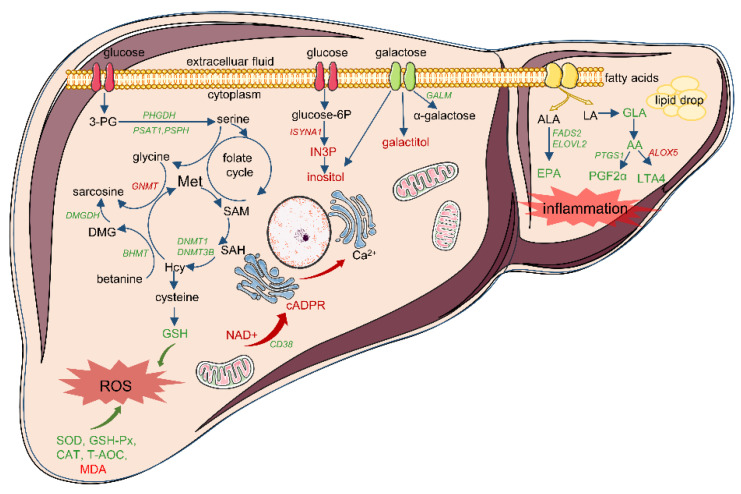
Summary of the DEGs and DMs were involved in a variety of metabolic abnormalities induced by oxidative stress in the liver of IUGR piglets. Abbreviations of DEGs identified by the transcriptomics analysis are shown in italics with red (high expression in IUGR group) or green (high expression in NBW group). DMs identified by metabolomics analysis are indicated either in red (highly expressed in the IUGR group) or green (highly expressed in the NBW group) color. Abbreviations: SAM, S-adenosylmethionine; SAH, S-adenosylhomocysteine; Hcy, homocysteine; LA, linolenic acid; ALA, alpha-linolenic acid; GLA, gamma-linolenic acid; EPA, eicosapentaenoic acid; NAD^+^, nicotinamide adenine dinucleotide; 3PG, 3-phospho glycerate; cADPR, cyclic ADP ribose; ROS, reactive oxygen species; SAM, S-adenosyl methionine; Hcy, homocysteine; IN3P, inositol 3-phosphate; Met, methionine; SAH, s-adenosylhomocysteine.

Moreover, excessive accumulation of sugar alcohol derivatives such as galactitol, sorbitol, and inositol was observed in the liver of IUGR piglets, all of which are involved in galactose metabolism. Galactose is normally converted into glucose through the Leloir pathway and then enters the glycolysis pathway or is stored as glycogen in the liver [42]. The key enzyme *GALM*-catalyzed galactose isomerization is the first step in this process [43]. Abnormal regulation of *GALM* in this metabolic process affects galactose conversion, resulting in the accumulation of galactose and its metabolic derivatives [44]. In our results, lower expression of *GLAM,* as well as higher levels of galactitol, sorbitol, and inositol, revealed that the galactose conversion pathway is blocked in the liver of IUGR piglets. It has been reported that accumulated galactitol may lead to free radical build-up, disrupting normal osmotic pressure and leading to redox imbalance in the liver [45]. In addition, inositol as a structural basis for secondary messengers in eukaryotic cells, is important in a variety of physiological processes such as intracellular growth, membrane biogenesis, and signal transmission [46]. Although inositol can be obtained from dietary supply, the amount of endogenous inositol synthesized in the liver is far higher than that ingested through diet. The synthesis of inositol begins with the isomerization of glucose-6-phosphate (glucose-6P) to inositol 3-phosphate (IN3P) by *ISYNA1*, followed by dephosphorylation to free inositol [47]. Therefore, in this study, the higher expression of *ISYNA1* mRNA may be the reason for the higher levels of inositol in livers of IUGR piglets. Clinically, altered concentration of inositol in plasma or urine is related to insulin resistance and diabetes, and a higher concentration of inositol has been proposed as a marker of IUGR in both humans [48] and pigs [49]. This is because inositol responds to insulin stimulation in the form of inositol phosphoglycans (IPGs) in liver tissue; decreased inositol availability or IPGs may lead to increased inositol circulation in insulin-resistant animals or humans [46,50]. Furthermore, it has been reported that increased inositol in fetuses with IUGR may reflect decreased glucose metabolic efficiency, which ultimately affects insulin secretion and fetal growth [51]. These results may suggest that galactose and its metabolites deteriorate liver energy homeostasis and cause oxidative stress in IUGR piglets (Figure 7).

## 5. Conclusions

In summary, the current study provides evidence that differences in gene expression and metabolites between the livers of IUGR and NBW piglets are involved in a variety of metabolic abnormalities, including mitochondrial dysfunction, imbalance of fatty acid composition, disruption to sources of one-carbon unit supply, and abnormal galactose conversion, thereby enhancing hepatic oxidative stress. Furthermore, oxidative stress status and hepatocyte damage were confirmed in the liver of IUGR piglets through the detection of antioxidant indices combined with morphological characteristics of the liver of IUGR piglets. Taken together, these data provide a detailed reference for understanding the metabolic characteristics of the liver in IUGR piglets and indicate that the mechanism of disordered metabolism in IUGR piglets might be associated with increased hepatic oxidative stress which ultimately affects the phenotypic characteristics of IUGR animals.

## Figures and Tables

**Table 1:** Major DEGs and DMs included in the integrated pathway related to oxidative stress.

Physiological Process	Pathway Term	DEGs	DMs
Mitochondrial dysfunction	Nicotinate and nicotinamide metabolism (ko00760); Oxytocin signaling pathway (ko04921); Aldosterone synthesis and secretion (ko04925); Calcium signaling pathway (ko04020)	*NT5E, CD38, SIRT4*, *NNMT, NT5C3A, ENPP3, BST1, NMRK2, LIPE, HSD3B, CDKN1A, CAMK1D, CAMK1, ADCY1, CACNB1, EGFR, CALML4, CACNB3, KCNJ2, PLCE1, PHKA2, ERBB2, GNAL, PLCD1, P2RX3, CXCR4*	NAD^+^, cADPR, AA, N’-Methyl-2-pyridone-5-carboxamide, 6-hydroxy-3-succinylpyridine
Imbalance of fatty acid composition	Biosynthesis of unsaturated fatty acids (ko01040); Linoleic acid metabolism (ko00591); alpha-Linolenic acid metabolism (ko00592); Arachidonic acid metabolism (ko00590)	*CYP2C, FADS2, CYP4A24, PTGS1, GGT5, ALOX5, CBR3, AKR1C2, ELOVL2, ACOT HSD17B12*	AA, GLA, EPA, 9,10-DiHOME, 12,13-DiHOME, FFA(18:4)
Disruption to sources of one-carbon unit supply	Glycine, serine and threonine metabolism (ko00260); Cysteine and methionine metabolism (ko00270)	*GATM, PSAT1, PHGDH, PSPH, DAO, GLYCTK, ALAS2, BHMT, DMGDH, AGXT2, PIPOX, GCAT, GNMT, GCLC, DNMT3B, LDHA, DNMT1, SRM, BCAT1*	N,N-Dimethylglycine, 5′-Deoxy-5′-(Methylthio) Adenosine
Abnormal galactose conversion	Galactose metabolism (ko00052); Inositol phosphate metabolism (ko00562); Fructose and mannose metabolism (ko00051)	*ISYNA1, PIK3C2G, GANC, GALM, MGAM2, LCT, MGAM, PFKFB4, TKFC, PI4KA, PLCE1, PLCD1, MTMR7*	galactitol, inositol, sorbitol, 3-hydroxyhippuric acid, mannitol, N-acetyl-D-galactosamine, GDP-fucose

## Data Availability

Not applicable.

## Supplementary Materials

The following supporting information can be downloaded at: www.mdpi.com/article/10.3390/biology11101430/s1. Table S1: Primer sequences of the DEGs identified by quantitative real-time polymerase chain reaction; Table S2: DMs between the IUGR and NBW piglets (VIP ≥ 1, *p* < 0.05, and absolute Log_2_FC ≥ 1); Table S3: DEGs between the IUGR and NBW piglets (log_2_Fold Change > 1 or <−1, *padj* < 0.05); Table S4: DEGs and DMs involved in the integrated pathways between the IUGR and NBW piglets.
